# Effects of two different perineal preparations before vaginal birth on maternal and neonatal infections and hospital resources: A randomized controlled trial

**DOI:** 10.18332/ejm/226734

**Published:** 2026-09-03

**Authors:** Tzu-Ying Huang, Li-Li Chen, Fu-Shiang Peng, Fang-Yeh Chu, Meei-Ling Gau

**Affiliations:** 1 Department of Nursing, Far Eastern Memorial Hospital, New Taipei City, Taiwan; 2 Department of Nurse-Midwifery and Women Health, National Taipei University of Nursing and Health Sciences, Taipei, Taiwan; 3 Department of Obstetrics and Gynecology, Far Eastern Memorial Hospital, New Taipei City, Taiwan; 4 Department of Clinical Pathology, Far Eastern Memorial Hospital, New Taipei City, Taiwan

**Keywords:** perineal preparation, postpartum infection, microbial flora, cost-effectiveness, randomized controlled trial

## Abstract

**INTRODUCTION:**

Perineal preparation during the second stage of labor may influence maternal and neonatal infection risk and healthcare resource utilization. Although povidone-iodine sterilization is traditionally used, microbial flora theory suggests that water cleansing may preserve normal flora without increasing infection while reducing workload and cost. This study compared water cleansing with povidone-iodine sterilization for perineal preparation at Far East Memorial Hospital, Taiwan, from 26 November 2024 to 5 June 2025.

**METHODS:**

A randomized controlled trial design was employed, including 143 mothers randomly assigned to either the water cleansing group (n=72) or the povidone-iodine sterilization group (n=71). Outcome measures included maternal and neonatal infection parameters (maternal postpartum temperature, C-reactive protein, REEDA scores, neonatal temperature, and neonatal oral flora colonization), along with cleansing time and medical costs. Data were analyzed using t-tests, chi-squared tests, and generalized estimating equation (GEE).

**RESULTS:**

GEE analysis revealed no significant differences between the two groups in maternal and neonatal infection indicators (REEDA, 95% CI: -0.53–0.18, p=0.337; maternal temperature, 95% CI: -0.06–0.21, p=0.278; neonate temperature, 95% CI: -0.12–0.19, p=0.636). The water group had significantly lower average medical costs than the povidone-iodine group (7.49 vs 110.92 TWD, t= -50.22, p<0.001), as well as a shorter cleansing time. Regarding neonatal flora colonization, the water group showed higher rates of normal flora (33.8% vs 15.5%) and lower rates of pathogenic bacteria (5.6% vs 12.7%, χ^2^=10.88, p=0.028).

**CONCLUSIONS:**

Perineal preparation with water was not associated with an increased risk of maternal or neonatal infection or adverse neonatal oral microbial colonization at birth and was associated with lower medical costs. These findings suggest that water may be a safe and cost-effective alternative to povidone-iodine for perineal preparation among women with low-risk pregnancies in hospitals with high episiotomy rates. Further longitudinal studies are needed to determine the effects of intrapartum povidone-iodine exposure on neonatal microbiome development and its potential long-term health consequences.

**CLINICAL TRIAL REGISTRATION:**

The study is registered on the official website of ClinicalTrials.gov

**IDENTIFIER:**

NCT06880445

## INTRODUCTION

Perineal antiseptic preparation is commonly performed before vaginal birth, particularly in countries where episiotomy remains prevalent, as a preventive measure to reduce the risk of maternal and neonatal infections^1^. However, with the increasing emphasis on evidence-based intrapartum care, current guidelines suggest that routine perineal antisepsis or sterilization before vaginal birth is unnecessary. Maintaining perineal cleanliness is considered sufficient, as routine attempts to achieve perineal sterility have not demonstrated additional maternal or neonatal benefits^2,3^.

A systematic review and meta-analysis evaluated the effectiveness of intrapartum vaginal chlorhexidine cleansing in preventing maternal and neonatal infections. The review included 11 randomized controlled trials comprising 20101 participants and examined outcomes including maternal and neonatal bacterial colonization and neonatal sepsis. No statistically significant differences were observed between the chlorhexidine and control groups for any of the primary outcomes. Overall, the review concluded that the available evidence remains insufficient to support the routine clinical use of intrapartum vaginal chlorhexidine cleansing^4^. Similarly, another study compared ‘clean’ versus ‘sterile’ childbirth models for low-risk births in two Italian hospitals^5^. Among 409 women included, no postpartum infections were observed in either group, suggesting that sterile techniques may not provide additional protection against infection compared to clean practices. Furthermore, the clean childbirth model offers several advantages, including reduced medicalization, lower healthcare costs, and closer alignment with the WHO’s recommendations for a positive and humanized childbirth experience.

Because maternal vaginal and perineal microbiota constitute one of the earliest sources of microbial exposure for newborns during vaginal birth, interventions that substantially reduce bacterial colonization may alter neonatal microbial acquisition^6^. Disruption of early microbial colonization has been associated with altered immune development and an increased risk of allergic and immune-mediated diseases, although the underlying causal mechanisms remain incompletely understood^6-8^. Furthermore, a recent study showed that intrapartum vaginal preparation with povidone-iodine may alter the maternal vaginal microbiome by reducing the relative abundance of Lactobacillus while increasing microbial diversity. Although povidone-iodine effectively decreases pathogenic bacterial contamination and reduces postoperative infectious morbidity, its potential impact on maternal vaginal microecology and subsequent neonatal microbial colonization requires further investigation^9^.

Although prior studies have suggested that water cleansing alone is sufficient for perineal preparation during labor, the reported low/no infection rates were primarily observed in settings where episiotomy was performed selectively and at relatively low rates. In contrast, in countries and healthcare institutions with a high prevalence of episiotomy, concerns remain regarding the potential risk of infection associated with perineal skin and tissue trauma caused by the surgical incision. A prospective multi-center cohort study in the UK investigated the prevalence of complications, primarily perineal wound infection, in 2021 women up to six weeks after childbirth. Women who undergo episiotomy have been reported to have a perineal wound infection rate of 13.1% (95% CI: 9.0–18.6) following spontaneous vaginal birth^10^. Therefore, healthcare providers may be reluctant to abandon antiseptic perineal preparation despite existing evidence supporting clean techniques. Accordingly, this study investigated the effects of two different perineal preparation methods during the second stage of labor – povidone-iodine antisepsis versus water cleansing – on maternal and neonatal postpartum infection rates, neonatal oral microbiota distribution, and overall medical costs in a Taiwanese medical center with a high episiotomy rate. By generating evidence-based research findings, this study seeks to inform clinical decision-making and support healthcare teams in adopting more ecologically sustainable and cost-effective care strategies while maintaining safety and effectiveness. Such strategies may further promote maternal and neonatal health and wellbeing, while enhancing the quality of childbirth care.

## METHODS

### Research design and setting

A parallel-group randomized controlled trial design was conducted to explore the effects of different perineal preparation methods (povidone-iodine sterilization versus water cleansing) during the second stage of labor on maternal and neonatal postpartum infection and cost-effectiveness. This study was conducted in the delivery room of a medical center at the Far East Memorial Hospital, Taiwan. The study was carried out from 26 November 2024 to 5 June 2025. The hospital routinely performed episiotomies, and the perineum was prepared with povidone-iodine prior to delivery.

A computer-generated randomization sequence was prepared by an independent statistician and concealed in sequentially numbered, opaque, sealed envelopes. Upon admission to the delivery room for childbirth, participants were assigned to either the experimental or control group by TYH (first author) according to the randomization sequence. Participants in the experimental group received perineal cleansing with water, whereas those in the control group underwent routine perineal sterilization with povidone-iodine in accordance with standard clinical protocol. All study interventions were administered by TYH. Data were collected by a trained research assistant who was blinded to group allocation.

All collected data were coded without indicating group allocation. To maintain blinding, statistical analyses were performed by an independent statistician who was unaware of the participants’ group assignments. In addition, laboratory measurements, including white blood cell count (WBC) and C-reactive protein (CRP), were analyzed by laboratory personnel who were blinded to group allocation. However, due to the nature of the intervention, participants and the intervention provider were not blinded. This RCT study has been registered at ClinicalTrials.gov (NCT06880445) on 30 November 2024.

### Participants

A convenience sample of low-risk pregnant women undergoing vaginal delivery was recruited. Participants were eligible if they met the following inclusion criteria: singleton pregnancy at term (≥37 weeks gestation) with vertex presentation, aged ≥18 years, conscious, able to communicate in Mandarin or Taiwanese and read Chinese, no antibiotic use during pregnancy, and no fetal abnormalities identified during prenatal examinations. Exclusion criteria included: rupture of membranes >18 h, fever, use of vacuum extraction delivery, shoulder dystocia, fetal distress, or third-degree or higher perineal lacerations.

Sample size was calculated using G*Power 3.1.9.7, with maternal and neonatal postpartum infection rate as the primary outcome variable. Considering that the study design used repeated measures ANOVA analysis, with power set at 0.8, α value of 0.05, effect size of 0.14, two-group comparison (number of groups=2), 3 repeated measures, and assuming correlation among repeated measures of 0.5, the calculation indicated 67 participants per group. Anticipating a dropout rate of approximately 20%, the target sample size was 168 participants.

### Instruments

Medical record data were used to collect demographic and obstetric data, including age, education level, weight, gestational age, neonatal weight, and epidural use.

The primary outcome of this study was maternal and neonatal postpartum infection rates. For maternal infection assessment, daily temperature, WBC, and C-reactive protein were collected^11^. Neonatal infection assessment included daily temperature evaluation and oral flora sampling and culture at birth. Additionally, the REEDA scale was used to assess perineal wound healing during the first three postpartum days. The scale evaluates five components, including redness, edema, ecchymosis, discharge, and approximation, with each item scored 0 to 3 points; higher total scores indicate poorer healing^12^. The REEDA scale has demonstrated marginal to good inter-rater reliability for assessing perineal wound healing. Inter-rater agreement was good for the discharge (κ=0.75–0.88) and redness (κ=0.46–0.66) items, marginal to good for the edema item (κ=0.16–0.46), and marginal for the ecchymosis item (κ= 0.25–0.42)^13^.

For cost-effectiveness evaluation, calculations were made from two perspectives: medical consumables and personnel costs. Medical consumables were standardized supplies used by the ward. The experimental group used regular tap water (37℃) provided by the delivery room as irrigation water, contained in unit-distributed irrigation bottles with a capacity of 300 mL for each cleansing session. According to a survey, the average water price in Taiwan in 2021 was 9.24 Taiwanese Dollars (TWD) per cubic meter^14^. The control group’s consumables included six sterile irrigation cotton swabs (5 TWD) and 200 mL povidone-iodine solution (100 TWD). For nursing personnel costs, calculations were based on the time (s) spent by nursing staff performing perineal cleansing, using the nursing personnel cost formula (sterilization time × nursing staff basic wage^15^) to calculate the expenditure amount.

### Intervention

Participants were randomly assigned to the experimental group (water cleansing) and control group (povidone-iodine sterilization). In the experimental group, the perineal area was cleaned with water, by means of warm water being slowly poured from an irrigation bottle, together with cleaning from above the perineal area toward the genital area. Cleansing with water included the area below the pubic symphysis, the external genitalia, and the upper third of the inner thighs. In the control group, cotton swabs were used to evenly apply or wipe povidone-iodine disinfectant on the perineal area (including below the pubic symphysis, external genitalia, and upper third of the inner thighs). The first cotton swab was used from the mons pubis upward to the lower abdomen, the second cotton swab from one leg’s inguinal area inward to outward to the anterior third of the thigh, the third cotton swab was used to disinfect the other side with the same method, and finally three more cotton swabs were used to separately disinfect the labia and vaginal opening to the anus, allowing the area to dry to achieve sterilization. Electronic stopwatches were used throughout to record operation time.

### Research procedure

The study was conducted after approval by the Institutional Review Board (IRB No. 113263-F). During hospitalization, eligible intrapartum women were identified by the researcher and randomly assigned to groups after obtaining written informed consent. To minimize bias in the intervention, all perineal preparations were performed by TYH, while data collection was conducted by a trained research assistant. Data collection included oral swab sampling of neonatal flora using sterile cotton swabs immediately after birth and REEDA scoring for postpartum wound assessment. Maternal and neonatal body temperatures and laboratory results (e.g. blood tests) were recorded from medical charts.

### Potential covariates

To minimize the potential influence of confounding variables on the intervention effects, potential covariates were collected, including demographic and obstetric characteristics (age, education level, maternal weight, gestational age, neonatal birth weight, and epidural analgesia use). In addition, baseline infection-related parameters, including pre-delivery WBC, CRP level, and body temperature, were collected.

### Statistical analysis

Statistical analyses were performed using SPSS version 30.0. Descriptive statistics were used to summarize demographic and obstetric characteristics, with continuous variables presented as means and standard deviations (SDs), and 95% confidence intervals (CIs), and categorical variables presented as frequencies and percentages. Independent-sample t-tests or chi-squared tests were used to compare demographic and obstetric data and baseline values (body temperature, prenatal CRP, WBC) between groups. Generalized estimating equations (GEEs) were used to evaluate the effects of the intervention on perineal wound healing and maternal and infant infection rates after adjusting for demographic, obstetric, and baseline variables. Neonatal bacterial species were analyzed using chi-squared tests, and t-tests were used to compare differences in medical costs between groups. A p<0.05 was considered statistically significant.

In terms of cost-effectiveness, manpower costs were calculated based on the time required for perineal cleansing and sterilization, while medical costs were analyzed according to the consumption of medical supplies (water, povidone-iodine, cotton swabs, and waste disposal).

Missing data were handled using complete-case analysis. Data were analyzed using the per-protocol population. Because post-randomization data on maternal and neonatal infection outcomes were unavailable for 25 randomized participants, a strict intention-to-treat analysis based on observed outcome data could not be performed.

### Ethics

This study was reviewed and approved by the IRB. Before implementation, communication with the clinical unit was completed, and informed consent was obtained. Participation was entirely voluntary, and all participants retained the right to withdraw at any time. Participant privacy and rights were fully respected throughout the research process, and all data were coded and anonymized for analysis.

## RESULTS

A total of 320 participants were initially assessed for eligibility. After excluding 152 individuals who did not meet the inclusion criteria or declined to participate, 168 participants were randomly assigned to either the experimental group (n=84) or the control group (n=84).

**Figure 1:**
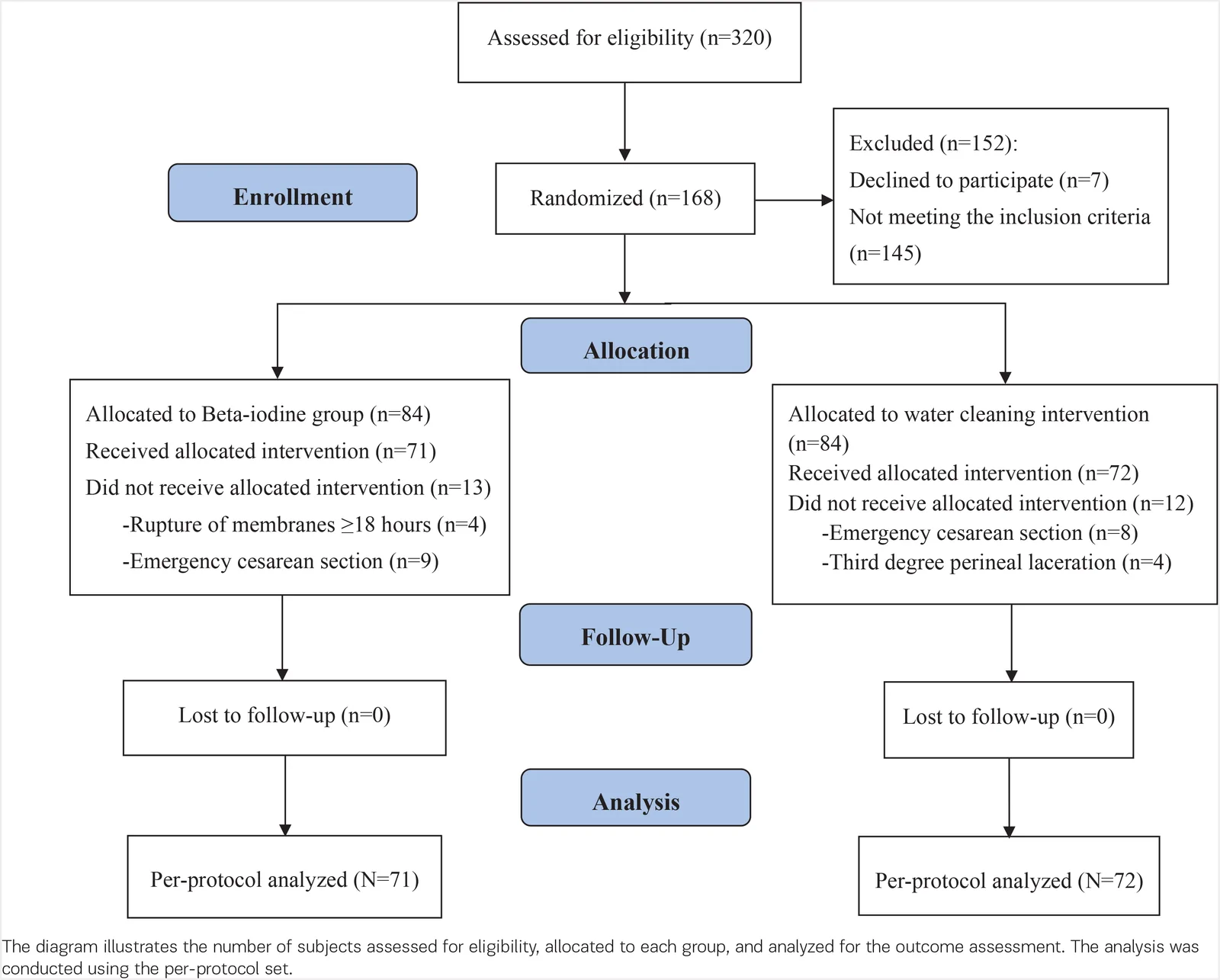
CONSORT diagram. Passage of participants through each trial stage in a randomized controlled trial of different perineal preparations before vaginal birth, Far Eastern Memorial Hospital, Taiwan, 2025

During the study, 13 participants from the experimental group and 12 from the control group did not receive the allocated interventions. The primary reasons for not receiving the allocated interventions were emergency cesarean birth (n=17), rupture of membranes for ≥18 h (n=4), and third-degree perineal laceration (n=4). Consequently, 143 postpartum women were included in the final analysis, comprising 72 participants in the experimental group and 71 participants in the control group (Figure 1).

A comparison of demographic and obstetric characteristics between participants included in the analysis (n=143) and those excluded after randomization (n=25) showed no significant differences in demographic characteristics, obstetric data, or neonatal Apgar scores (p>0.05) (Supplemental file Table S1).

### Baseline characteristics

The mean age of the participants was 33.6 ± 4.3 years, and the mean maternal weight at admission was 70.1 ± 9.8 kg. Most participants had a college-level education (69.9%). The mean gestational age at delivery was 38.9 ± 0.9 weeks, and the mean neonatal birth weight was 3173.0 ± 305.8 g. The epidural anesthesia rate was 81.8%. There were no significant differences between the two groups in demographic characteristics, obstetric data, or neonatal Apgar scores (p>0.05), indicating homogeneity between the samples (Table 1).

**Table 1:** Baseline demographic and obstetrics data of participants in a randomized controlled trial of two different perineal preparations before vaginal birth, Far Eastern Memorial Hospital, Taiwan, 2025 (N=143)

*Variables*	*Overall* *(N=143)* *n (%)*	*Control* *(N=71)* *n (%)*	*Experimental* *(N=72)* *n (%)*	*χ^2^/t*	*p*
**Age** (years), mean (SD) [95% CI]	33.6 (4.3) [32.9–34.3]	33.5 (3.8) [33.6–34.0]	33.6 (4.8) [32.5–34.7]	0.18^a^	0.86
**Pre-labor weight** (kg), mean (SD) [95% CI]	70.1 (9.8) [68.5–71.7]	70.0 (9.6) [67.7–72.3]	70.2 (10.0) [67.9–72.6]	0.14^a^	0.89
**Education level**				0.91^b^	0.82
Junior school	2 (1.4)	1 (1.4)	1 (1.4)		
High school	15 (11.0)	6 (8.5)	9 (12.5)		
Bachelor’s	100 (69.9)	52 (73.2)	48 (66.7)		
Graduate	26 (18.2)	12 (16.9)	14 (19.4)		
**Gestational weeks**, mean (SD) [95% CI]	38.9 (0.9) [38.8–39.1]	39.0 (0.9) [38.8–39.2]	38.8 (0.9) [38.6–39.0]	-1.02^a^	0.31
**Epidural**	117 (81.8)	55 (77.5)	62 (86.1)	1.80^b^	0.18
**Neonate weight** (g), mean (SD) [95% CI]	3173.0 (305.8) [3122.4–3223.6]	3186.3 (280.7) [3119.8–3252.7]	3159.9 (330.2) [3082.3–3237.5]	-0.51^a^	0.61
**Apgar (1 min, ≥7)**	137 (95.8)	68 (95.8)	69 (95.8)	0.00^b^	0.99
**Apgar (5 min, ≥7)**	143 (100.0)	71 (100)	72 (100)	-	-

a t-test.

b Pearson chi-squared. Control: povidone-iodine group. Experimental: water group.

### Primary outcomes

*Maternal and neonate infection indicators*

Regarding maternal infection indicators, the results showed no significant differences between the groups in terms of physiological parameters, including intrapartum temperature during the second stage of labor (p=0.172), daily postpartum temperature changes (p=0.074–0.285), antenatal WBC (p=0.297), and CRP levels (antenatal p=0.096, postnatal p=0.368). Similarly, neonatal temperature monitoring revealed no significant differences in rectal temperatures between the two groups from birth to the third postpartum day (p=0.147–0.444) (Table 2).

**Table 2:** Univariate analysis of the comparison of infection indices between groups in a randomized controlled trial of two different perineal preparations before vaginal birth, Far Eastern Memorial Hospital, Taiwan, 2025 (N=143)

*Variables*	*Overall* *(N=143)* *Mean ± SD*	*Control* *(N=71)* *Mean ± SD*	*Experimental* *(N=72)* *Mean ± SD*	*t*	*p*
**REEDA score**					
PP1	1.45 ± 1.06	1.54 ± 1.11	1.37 ± 1.02	-0.95	0.345
PP2	0.75 ± 0.75	0.80 ± 0.75	0.70 ± 0.74	-0.79	0.433
PP3	0.20 ± 0.40	0.27 ± 0.45	0.13 ± 0.34	-2.13	0.035*
**Maternal BT** (°C)					
At birth	37.32 ± 0.50	37.36 ± 0.50	37.28 ± 0.51	-0.95	0.172
PP1	36.39 ± 0.32	36.43 ± 0.32	36.35 ± 0.31	-1.46	0.074
PP2	36.26 ± 0.27	36.28 ± 0.29	36.24 ± 0.25	-1.02	0.155
PP3	36.29 ± 0.24	36.28 ± 0.23	36.30 ± 0.24	0.57	0.285
**NB BT** (°C)					
At Birth	37.53 ± 0.51	37.51 ± 0.49	37.55 ± 0.54	0.46	0.324
PP1	36.81 ± 0.17	36.80 ± 0.16	36.81 ± 0.18	0.14	0.444
PP2	36.75 ± 0.17	36.74 ± 0.16	36.77 ± 0.18	0.88	0.190
PP3	36.68 ± 0.14	36.69 ± 0.15	36.67 ± 0.12	-1.06	0.147
**Antenatal WBC**	9.89 ± 2.68	9.77 ± 2.70	10.01 ± 2.66	0.53	0.297
**CRP** (antenatal)	0.53 ± 0.72	0.46 ± 0.34	0.61 ± 0.95	1.31	0.096
**CRP** (postnatal)	4.30 ± 3.06	4.39 ± 3.32	4.22 ± 2.80	-0.34	0.368
	***n (%)***	***n (%)***	***n (%)***		
**Bacterial species**				χ^2^=10.88	0.028*
No growth	66 (46.5)	33 (53.5)	33 (38.4)		
*Lactobacillus*	26 (18.3)	11 (15.5)	15 (21.1)		
*Bifidobacterium*	2 (1.4)	2 (2.8)	0 (0)		
Normal flora	35 (24.6)	11 (15.5)	24 (33.8)		
*Escherichia coli*	13 (9.2)	9 (12.7)	4 (5.6)		

Control: povidone-iodine group. Experimental: water group. REEDA: Redness, Edema, Ecchymosis, Discharge, and Approximation. NB: newborn. BT: body temperature. PP: postpartum. WBC: white blood count. CRP: C-reactive protein.

* p<0.05.

Further analysis using the GEE revealed that, after controlling for maternal age, gestational age, prenatal WBC, prenatal CRP, postpartum CRP, and maternal body temperature, there was no significant difference in REEDA scores between the two perineal preparation methods (B= -0.17, p=0.337). Regarding the time variable, compared with postpartum day 1, REEDA scores significantly decreased on postpartum day 2 (B= -0.74, p<0.001) and showed an even greater decrease on postpartum day 3 (B= -1.28, p<0.001) (Table 3).

**Table 3:** GEE analysis of the differences of REEDA score, maternal temperature, and neonatal temperature between groups in a randomized controlled trial of two different perineal preparations before vaginal birth, Far Eastern Memorial Hospital, Taiwan, 2025 (N=143)

Variables	B	SE	95% CI	Wald χ^2^	p
**REEDA score**					
**Group** (ref: Control) Experimental	-0.17	0.18	-0.53–0.18	0.92	0.337
**Days** (ref: PP day 1)					
PP day 3	-1.28	0.11	-1.49 – -1.06	134.08	<0.001*
PP day 2	-0.74	0.09	-0.92 – -0.56	66.41	<0.001*
**Antenatal WBC**	-0.01	0.02	-0.05–0.03	0.49	0.483
**Antenatal CRP**	0.02	0.05	-0.09–0.12	0.08	0.774
**Postnatal CRP**	0.02	0.02	-0.01–0.06	1.49	0.222
**Postnatal BT** (°C)	-0.05	0.14	-0.32–0.22	0.15	0.702
**Maternal temperature** (°C)					
**Group** (ref: Control) Experimental	0.07	0.07	-0.06–0.21	1.18	0.278
**Days** (ref: at birth)					
PP day 3	-0.25	0.05	-0.35 – -0.15	24.15	<0.001*
PP day 2	-0.25	0.05	-0.35 – -0.15	23.33	<0.001*
PP day 1	-0.10	0.05	-0.19 – -0.01	431	0.038*
**Antenatal WBC**	-0.01	0.01	-0.02–0.01	1.42	0.234
**Antenatal CRP**	-0.03	0.02	-0.08–0.02	1.74	0.187
**Postnatal CRP**	0.01	0.01	0.00–0.03	4.28	0.039*
**Neonate temperature** (°C)					
**Group** (ref: Control) Experimental	0.04	0.08	-0.12–0.19	0.22	0.636
**Days** (ref: at birth)					
PP day 3	-0.64	0.07	-0.77 – -0.50	85.06	<0.001*
PP day 2	-0.59	0.07	-0.73 – -0.45	65.52	<0.001*
PP day 1	-0.55	0.06	-0.68 – -0.43	74.60	<0.001*
**Antenatal WBC**	0.01	0.01	0.00–0.02	6.49	0.011*
**Antenatal CRP**	0.01	0.02	-0.03–0.04	0.11	0.742
**Postnatal CRP**	0.01	0.01	-0.00–0.02	2.60	0.107
**Maternal temperature** (°C)	0.17	0.05	0.08–0.26	13.05	<0.001*

Control: povidone-iodine group. Experimental : water group. SE: standard error. CI: confidence interval. REEDA : Redness, Edema, Ecchymosis, Discharge, and Approximation. NB: newborn. BT: body temperature. PP: postpartum. WBC: white blood count. CRP: C-reactive protein.

* p<0.05.

For maternal temperature, the perineal preparation method showed no significant effect (B=0.07, p=0.278). Compared with the day of delivery, maternal temperature significantly decreased on postpartum day 1 (B= -0.10, p=0.038), with further decreases observed on day 2 (B= -0.25, p<0.001) and day 3 (B= -0.25, p<0.001). Among the controlled variables, only postpartum CRP was a significant predictor (B=0.01, p=0.039). Other factors, such as maternal age, prenatal WBC, and prenatal CRP, showed no significant effect on maternal temperature (p>0.05) (Table 3).

Regarding neonatal temperature, results indicated that the perineal preparation method had no significant effect (B=0.04, p=0.636). Neonatal body temperature naturally declined and stabilized as part of the normal physiological adaptation after birth. However, both maternal temperature (B=0.17, p<0.001) and prenatal WBC (B=0.01, p=0.011) were found to significantly influence neonatal temperature (Table 3).

In addition, the neonatal oral bacterial culture results showed that no bacterial growth was most common (46.5%), followed by normal flora (24.6%), Lactobacillus (18.3%), and Escherichia coli (9.2%). Chi-squared analysis of bacterial distribution revealed that the control group had a higher proportion of sterile samples (53.5% vs 38.4%), whereas the experimental group had higher detection rates of normal flora and Lactobacillus. The overall distribution of bacterial species showed a significant difference (χ²=10.88, p=0.028) (Table 2).

*Cost effectiveness*

The results showed that the total cost for the water cleansing group was TWD 6.05, which was significantly lower than that of the povidone-iodine sterilization group (TWD 112.45). The time required for nurses to perform perineal preparation was also shorter in the water cleansing group (39.60 ± 2.05 s) compared to the povidone-iodine group (66.95 ± 10.37 s, p<0.001).

Based on an estimated nursing wage rate of TWD 0.0828 per second^15^, the average nursing manpower cost was TWD 5.54 for the povidone-iodine group and TWD 3.28 for the water cleansing group (Table 4). Regarding medical material consumption, the povidone-iodine group incurred a total cost of TWD 106.91 per person for cotton swabs, solution use, and waste disposal, whereas the water cleansing group averaged only TWD 2.77 per person (Table 4).

**Table 4:** Comparison of perineal preparation time, nursing costs, and medical expenses between groups in a randomized controlled trial of two different perineal preparations before vaginal birth, Far Eastern Memorial Hospital, Taiwan, 2025 (N=143)

*Variables*	*Control* *(N=71)* *Mean ± SD*	*Experimental* *(N=72)* *Mean ± SD*	*t*	*p*
**Perineal preparation time** (s)	66.95 ± 10.37	39.60 ± 2.05	-21.22	<0.001*
**Nursing costs** (TWD)	5.54 ± 0.89	3.28 ± 0.17	-21.22	<0.001*
**Medical expenses** (TWD)				
Beta iodine or water	100	2.77	-	-
Cotton swab	5	0	-	-
Waste disposal fee	1.91	0	-	-
**Total costs** (TWD)	112.45 ± 0.89	6.05 ± 0.17	-996.62	<0.001*

Control: povidone-iodine group. Experimental: water group. Total costs: nursing costs + medical expenses + waste disposal fee. TWD: 1000 Taiwanese Dollars about US$31.

* p<0.05.

## DISCUSSION

This study found no differences between perineal preparation methods in maternal and neonatal body temperature, maternal CRP levels, or the extent of perineal wound healing. All maternal body temperatures remained within the normal range, indicating that routine use of water cleansing does not increase infection risk in low-risk populations. Furthermore, neonatal temperatures remained stable and within normal limits, suggesting that the different perineal preparation methods did not adversely affect neonatal physiological stability.

These results are consistent with previous studies^4,5,9,16^, which reported that water cleansing does not increase infection rates among low-risk parturients and may reduce skin irritation and the potential adverse effects associated with excessive cleansing. From a microbiological and skin-physiological perspective, water cleansing may help maintain the normal bacterial flora of the perineal and vaginal areas^17^. Beneficial bacteria, such as *lactobacilli*, contribute to maintaining an acidic environment through the production of lactic acid and hydrogen peroxide, which may inhibit the growth of potential pathogens, including *Staphylococcus aureus* and *E. coli*^18^. In contrast, antiseptics have broad bactericidal effects that may reduce both potentially pathogenic and commensal bacteria^8,18^. In our study, the povidone-iodine group showed a slightly higher proportion of potentially pathogenic bacterial species, whereas the water cleansing group had a higher proportion of samples showing normal flora or no bacterial growth, suggesting that water cleansing may be associated with a more favorable bacterial profile based on culture results.

Previous studies^9,19^ have shown that newborns are exposed to maternal vaginal and perineal bacteria during birth. Excessive sterilization of these areas may alter normal bacterial exposure during delivery. Our study further suggests that, without compromising neonatal thermoregulatory stability, water cleansing may be a potential approach for maintaining normal bacterial flora during childbirth. Moreover, the skin is the first line of defense against infection. The literature indicates that povidone-iodine may cause mild skin irritation, dryness, or cracking, potentially weakening this physical barrier. Water cleansing is mild and non-irritating, which helps maintain skin integrity^17^.

GEE analysis showed that after adjusting for confounders, water cleansing is feasible and safe for maintaining maternal and neonatal physiological stability. Although postpartum CRP was a significant predictor of maternal temperature, its variation primarily reflects maternal inflammatory responses and is not directly attributable to the type of perineal preparation. REEDA scores also exhibited a time-dependent decline, supporting an association between perineal wound healing and time and being consistent with previous findings that the presence and balance of vaginal and perineal bacteria may influence wound healing processes^20^.

Regarding resource utilization, water cleansing required significantly less time and had a much lower overall consumables cost, the latter difference largely attributable to the price of povidone-iodine solution and sterile cotton swabs. Nursing manpower costs were also reduced due to the simplified procedure, which can help alleviate clinical workload and improve care efficiency. These findings align with previous studies advocating process simplification to enhance cost-effectiveness^21^. Over recent decades, attention to appropriate care has grown globally (e.g. the ‘Choosing Wisely’ campaign and the establishment of ‘do-not-do’ recommendations), aiming to reduce low-value medical care^22^. Therefore, pre-delivery perineal cleansing with water can be considered an evidence-based, precision-medicine approach that ensures safety while avoiding unnecessary medical waste.

### Limitations

Although this study employed a randomized controlled design and standardized procedures, several limitations warrant cautious interpretation of the results. First, the sample was drawn from a single medical center in northern Taiwan, which limits generalizability; external validity should be improved in future studies. Second, the observation period was limited to the first three postpartum days. Consequently, long-term maternal and neonatal outcomes, including delayed postpartum infections, perineal wound healing, and the stability and development of the neonatal microbiome, could not be evaluated. Furthermore, perineal wound healing was assessed using the REEDA scale during the early postpartum period only. Because physiological wound healing progresses gradually over time, the selected assessment time point may not have been sufficiently sensitive to detect subtle differences between groups. Therefore, the absence of significant differences in REEDA scores should be interpreted with caution. In addition, although the assessor received standardized training and used a structured assessment tool, REEDA scoring involves a degree of subjective clinical judgment, and measurement bias cannot be entirely excluded. The lack of double-blinding may have further increased the potential for observer bias. However, most outcome measures were objective physiological variables (e.g. temperature, CRP, bacterial culture), which mitigates threats to internal validity.

Third, only a single neonatal oral sample was collected immediately after birth. Consequently, temporal changes in microbial colonization and the persistence of any microbiological differences between the study groups could not be evaluated.

### Future research

Future studies should consider multicenter, larger sample designs encompassing different regions and levels of care to enhance generalizability and applicability. Follow-up could be extended to one week or one month postpartum to evaluate long-term infection control, neonatal health outcomes, and microbiome development. Additionally, incorporating qualitative interviews would help explore maternal acceptability, comfort, and satisfaction with different perineal care methods.

## CONCLUSIONS

This randomized controlled trial compared two perineal preparation methods during the second stage of labor – water cleansing versus povidone-iodine sterilization – with respect to maternal infection risk, neonatal oral bacterial colonization, and medical costs. The results indicate that water cleansing, without adversely affecting maternal physiological indicators or infection control, could substantially reduce consumable and personnel costs and offers advantages in ease of use and clinical scalability. Regarding neonatal bacterial colonization, water cleansing was not associated with adverse bacterial culture findings. Therefore, for cases without infection risk, water cleansing could potentially be considered as a routine perineal management option, accompanied by reinforced hand hygiene and aseptic techniques to ensure infection control quality. These findings should be incorporated into nursing and midwifery education to strengthen learners’ knowledge and clinical application regarding intrapartum vaginal preparation, infection prevention, and resource management.

## Supplementary Material



## Data Availability

The data supporting this research are available from the authors on reasonable request.
