# Supplementary material for: Effects of two different perineal preparations before vaginal birth on maternal and neonatal infections and hospital resources: A randomized controlled trial
Source: Eur J Midwifery. 2026 Sep 3;10:10.18332/ejm/226734. doi: 10.18332/ejm/226734 (PMC13539763; doi:10.18332/ejm/226734)
Supplement: Supplementary file 1 [file EJM-10-39-s1.pdf]

EUROPEAN JOURNAL
OF MIDWIFERY

**Supplementary file**

© 2026 Huang T. Y. et al.

**DOI:**
10.18332/ejm/226734

The content has been provided by the author(s) and has not been reviewed, verified, or endorsed by European Publishing. It may not have undergone peer review. The views, opinions, and recommendations expressed are solely those of the author(s) and do not necessarily reflect the position of European Publishing. European Publishing accepts no responsibility or liability for any consequences arising from the use of, or reliance on, this content.

***Supplemental Table S1 . Comparison of demographic and obstetrics data of enrolled participants and participants lost to follow-up in a randomized controlled trial of two different perineal preparations before vaginal birth, Far Eastern Memorial Hospital, Taiwan, 2025 (N = 168)***

| Variables | Enrolled (*n*=143) *n* (%) | Lost follow-up (*n* = 25) *n*(%) | $\chi^2/Z$ | *p* |
|---|---|---|---|---|
| **Age** (years), mean (SD) [95% CI] | 33.6 (4.3) [32.9-34.3] | 34.8 (4.9) [32.5-34.7] | -1.18[a] | .24 |
| **Pre-labor weight** (kg), mean (SD) [95% CI] | 70.1 (9.8) [68.5-71.7] | 70.8 (11.4) [67.9-72.6] | -0.80[a] | .94 |
| **Education** | | | 2.86[b] | .41 |
| Junior school | 2(1.4) | 0(0) | | |
| High school | 15(11.0) | 2(8.0) | | |
| Bachelor | 100(69.9) | 15(60.0) | | |
| Graduate | 26(18.2) | 8(32.0) | | |
| **Gestational weeks** (weeks), Mean (*SD*) [95% CI] | 38.9 (0.9) [38.8-39.1] | 39.1 (1.1) [38.6-39.0] | -1.11[a] | .27 |
| **Epidural** | 117 (81.8) | 23 (92.0) | 1.59[b] | .21 |
| **Neonate weight** (grams), mean (*SD*) [95% CI] | 3173.0 (305.8) [3122.4-3223.6] | 3249.2 (330.2) [3082.3-3237.5] | -.90[a] | .37 |
| **Apgar (1 min, ≧7)** | 137 (95.8) | 22 (88.0) | 2.56[b] | .11 |
| **Apgar (5 min, ≧7 )** | 143 (100.0) | 25 (100) | -- | -- |

Note: [a] Mann-Whitney test; [b] Pearson Chi-Square
